# Developmental stages and molecular phylogeny of *Hepatozoon fitzsimonsi* (Dias 1953) (Adeleorina: Hepatozoidae) in tortoises *Stigmochelys pardalis* (Cryptodira: Testudinidae) and ticks of the genus *Amblyomma* (Acari: Ixodidae) from South Africa

**DOI:** 10.1186/s13071-024-06398-z

**Published:** 2024-07-20

**Authors:** Lehlohonolo S. Mofokeng, Edward C. Netherlands, Nico J. Smit, Courtney A. Cook

**Affiliations:** 1 Water Research Group, Unit for Environmental Sciences and Management, North-West University, Potchefstroom, 2531 South Africa; 2 Department of Zoology and Entomology, University of the Free State, Private Bag X13, Phuthaditjhaba, 9866 South Africa; 3 Department of Zoology and Entomology, University of the Free State, PO Box 339, Bloemfontein, 9300 South Africa

**Keywords:** *Amblyomma* ticks, Developmental stages, *Hepatozoonfitzsimonsi*, Sporogony, Phylogeny, Tortoises, Merogony, Haemogregarine

## Abstract

**Background:**

*Hepatozoon fitzsimonsi* (Dias, 1953) is a frequently found haemogregarine of southern African tortoises. At the time of this species’ reassignment from the genus *Haemogregarina* to *Hepatozoon*, developmental stages such as sporocysts and sporozoites were observed in ticks associated with *H. fitzsimonsi* parasitised and non-parasitised tortoises. It was thus suggested that ticks may act as the potential vectors for this parasite. However, this earlier research was unable to confirm the identity of these sporogonic stages using molecular markers. In a separate study aimed at identifying tick species parasitising South African reptiles and molecularly screening these for the presence of *Hepatozoon*, that study identified *H. fitzsimonsi* in tortoise-associated ticks. Thus, the present study aimed to revisit the potential of ticks to act as vectors for *H. fitzsimonsi* in tortoises using a combined microscopy and molecular approach.

**Methods:**

Specimens of *Kinixys natalensis*, *Kinixys spekii*, *Kinixys zombensis* and *Stigmochelys pardalis* were collected from Bonamanzi and Ndumo Game Reserve, South Africa. Upon capture, animals were examined for ticks, and these were collected along with blood and other tissues. Adult ticks were dissected and visceral impression slides were prepared along with thin blood and tissue smears on clean microscope slides. Smears and impression slides were stained with Giemsa, screened and micrographs of parasites were captured. Two primer sets were employed to target fragments of the 18S rRNA gene of parasites found in both tortoises and ticks and the resulting sequences were then compared with other known *H. fitzsimonsi* and haemogregarine sequences from the GenBank database.

**Results:**

Peripheral blood gamont and liver merogonic stages were observed in *S. pardalis*, while the sporogonic stages were observed in the haemocoel of *Amblyomma* ticks. Gamont and sporocyst stages compared morphologically with previous descriptions of *H. fitzsimonsi*, identifying them as this species. Phylogenetic analysis revealed that the blood and tick sequences obtained in this study clustered in a monophyletic clade comprising known *H. fitzsimonsi*.

**Conclusions:**

The present study provides further support for ticks acting as the vectors of *H. fitzsimonsi* by molecularly identifying and linking observed developmental stages in tortoises (*S. pardalis*) with those in the invertebrate host (*Amblyomma* spp.).

**Graphical Abstract:**

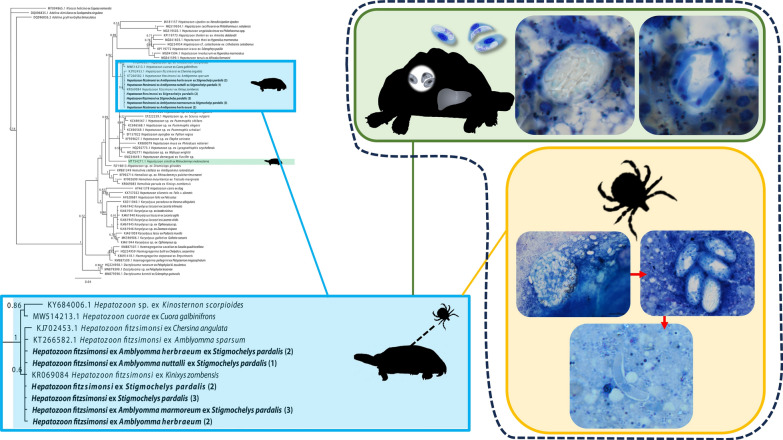

## Background

Haemogregarines (Apicomplexa: Adeleorina) are commonly encountered protozoan parasites of erythrocytes or leukocytes and are frequently described from a wide variety of vertebrates including amphibians and reptiles [1–5]. Even though this group of parasites contains several genera, such as *Hemolivia* Petit et al. 1990, *Haemogregarina* Danilewsky 1985 and *Karyolysus* Labbé 1894, the genus *Hepatozoon* Miller 1908 appears to be the most prevalent in reptiles [3, 6]. However, even with the increase in research on these parasites in the last decade, knowledge on the diversity and systematics of these apicomplexans is still lacking and remains contentious [4, 7]. Based on relationships of these haemogregarine genera inferred using the 18S ribsomal RNA (rRNA) gene, *Hepatozoon* remains paraphyletic [5–11]. This lead Karadjian et al. [12] to erect a new genus *Bartazoon* Karadjian et al., 2015, to resolve this issue. Species of *Hepatozoon* display a heteroxenous life cycle, requiring a vertebrate host in which asexual reproduction occurs, as well as an invertebrate host in which sexual reproduction occurs [6, 12, 13]. Invertebrate hosts range from haematophagous insects to acarine hosts such as ticks and mites [12]. For transmission to occur between the parasitised vector and the vertebrate host, ingestion of this vector by the vertebrate is required or ingestion of tissue cysts in a vertebrate host is required [1, 12]. Karadjian et al. [12] proposed that *Bartazoon* would comprise species vectored by haematophagous insects, whilst those species vectored by acarine hosts would remain as *Hepatozoon*. However, *Bartazoon* has up to now not been widely accepted as the monophyly of this genus is not well supported [7, 11, 14].

*Hepatozoon fitzsimonsi* (Dias, 1953), a haemogregarine of southern African tortoises, along with other *Hepatozoon* species of amphibians and reptiles, was one of the species proposed to be a member of *Bartazoon* [12]. This, as such, suggested the potential vector of this parasite to be a biting insect. However, research done on this parasite persistently observed a close association with tortoises and ticks [3, 15], the preceding study observing what appeared to be sporogonic stages including sporocysts and sporozoites in tick haemocoel from infected and uninfected tortoises. Even though Cook et al. [3] attempted to molecularly identify these tick stages, it was unsuccessful. Recently, two molecular screening studies [11, 16], both screening ticks collected from reptiles in Kenya and South Africa for tick-borne pathogens and *Hepatozoon*, respectively, identified *H. fitzsimonsi* in ticks. In both studies unengorged ticks were used. With these previous findings in mind, the present study aimed to revisit the potential that ticks can act as vectors for *H. fitzsimonsi* in tortoises, by (i) collecting blood/tissue and ticks from tortoises, (ii) screening both microscopically for the presence of blood, merogonic and sporogonic stages, respectively, and (iii) molecularly characterising these stages to determine if they are that of *H. fitzsimonsi*. The present study thus provides further support for ticks acting as the vectors of *H. fitzsimonsi* based on observation of its life cycle stages in tortoises (*Stigmochelys pardalis*) as well as in the invertebrate host (*Amblyomma* spp.).

## Methods

### Tortoise and tortoise tissue collection, and preparation of blood and other tissue slides

Tortoises were collected during 2016–2017 from Bonamanzi and Ndumo Game Reserve, KwaZulu-Natal (KZN), and identified to species level using field guides [17–19]. As these animals are protected by law, permits did not allow for euthanasia; however, in one case a tortoise was found recently dead, the result of a roadkill incident.

Blood was collected from the subcarapacial sinuses [20] and thin blood smears were prepared, and allowed to air dry before being fixed in absolute methanol and stained in a solution of Giemsa stain (FLUKA, Sigma-Aldrich, Steinheim, Germany) for 20 min [3]. Once stained and dried, these were stored in a dustproof container for later screening. A small volume of blood was preserved in 70% ethanol for molecular work. Samples from the roadkill individual were taken from the kidney, heart, liver, lung, and spleen during dissection. Impression and squash smears were prepared on clean microscope slides, and these fixed and stained following the same method as described for the blood smears. A small section of each organ was preserved in 70% ethanol.

### Tick collection, with preparation of tick impression slides

Ticks present on tortoises at the time of blood collection were carefully removed using forceps to ensure that the hypostome remained intact with minimal damage to the tick. Ticks were placed in plastic tubes and labelled according to the individual tortoise from which they were collected. Ticks were allowed to digest their blood meals for approximately 10–20 days [13, 21]. Thereafter, adult ticks were dissected according to Edwards et al. [22], and visceral impression slides were prepared on clean microscope slides. The remaining tick and its viscera were immediately fixed in 70% ethanol and stored in a −20 °C freezer until further molecular analysis. Slides were allowed to dry and followed the same fixing and staining protocol as that of the blood and organ smears. Morphological identification to species level was done with the aid of a Nikon AZ100M microscope and guidelines for tick identification as provided by Theiler [23] and Theiler and Salisbury [24].

### Microscopical screening of tortoise blood and tissue smears and tick impression slides

Stained blood smears were screened at 100 × oil immersion objective, and micrographs of parasites were taken on a calibrated Nikon Eclipse E800 compound microscope (Nikon, Amsterdam, the Netherlands) using the imaging software NIS Elements. All measurements were in micrometres (µm) unless otherwise indicated. Measurements comprised the parasite’s length (including recurved tail when present) and width within its parasitophorous vacuole (PV), and the parasite’s nucleus length and width [3]. Subsequently, the morphometric data of parasites were used in comparison to previous descriptions of *H. fitzsimonsi*.

Impression slides were screened using the 20×, 60× and 100× oil immersion objective on a calibrated Nikon Eclipse E800 compound microscope (Nikon, Amsterdam, the Netherlands) using the imaging software NIS Elements. Micrographs of sporogonic stages were taken using both the 20× and 100× objectives, depending on the size of the parasite stage. Sporogonic stages were identified and measured according to Cook et al. [3], with the aid of the ImageJ version 1.47 software program (Wayne Rasband National Industries of Health, USA) [25] (http://imagej.nih.gov/ij) and subsequently compared with those observed in ticks from Cook et al. [3]. The number of sporozoites within each sporocyst were estimated by counting their nuclei [3].

### DNA extraction, PCR and phylogenetic analysis

Genomic DNA was extracted from the blood and liver samples of tortoises, as well as from visceral samples of ticks using the KAPA Express Extract Kit (Kapa Biosystems, Sigma-Aldrich). Once extracted, DNA was used for polymerase chain reaction (PCR) amplification, amplifying approximately the full 18S rRNA gene in two fragments for the tortoise samples by using a combination of primer sets. The first fragment, approximately 930 nt in length, was amplified using primer set HAMF 5′-GCCAGTAGTCATATGCTTGTC-3′ [26] and HepR900 5′-CAAATCTAAGAATTTCACCTCTGAC-3′ [27]. The second fragment, approximately 1400 nt in length, was amplified using primer set HepF300 5′-GTTTCTGACCTATCAGCTTTCGACG-3′ [27] and 2868 5′-TGATCCTTCTGCAGGTTCAC-3′ [28, 29].

Conditions for PCR of both fragments were as follows: initial denaturation at 95 °C for 3 min, followed by 35 cycles, entailing a 95 °C denaturation for 30 s, annealing at 61 °C for 30 s with an end extension at 72 °C for 2 min, and following the cycles a final extension of 72 °C for 10 min [30].

Difficulties were encountered using the above primer sets and protocols for ticks (only that for *Amblyomma marmoreum* was partially successful), as such for the tick visceral samples, fragments of the 18S rRNA gene were amplified using the primer set HepF300 5′-GTTTCTGACCTATCAGCTTTCGACG-3′ and HepR900 5′-CAAATCTAAGAATTTCACCTCTGAC-3′, targeting a fragment of approximately 600 nt.

Conditions for PCR were as follows: initial denaturation at 95 °C for 3 min, followed by 35 cycles, entailing a 95 °C denaturation for 30 s, annealing at 60 °C for 30 s with an end extension at 72 °C for 1 min, and following the cycles a final extension of 72 °C for 10 min as detailed according to previous methods [31].

PCR reactions were performed with volumes of 25 μL, using 12.5 μL Thermo Scientific DreamTaq PCR master mix (2×) (final concentration: 2× DreamTaq buffer, 0.4 mM of each dNTP and 4 mM MgCl2), 1.25 μL (10 μM) of each of the primer sets mentioned above, and at least 25 ng DNA. The final reaction volume was made up with PCR-grade nuclease-free water (Thermo Scientific). Reactions were undertaken in a Bio-Rad C1000 Touch™ Thermal Cycler PCR machine (Bio-Rad, Hemel Hempstead, UK). Resulting amplicons were visualized under ultraviolet light on a 1% agarose gel stained with gel red using a Bio-Rad GelDoc^™^ XR + imaging system (Bio-Rad, Hemel Hempstead, UK). PCR products from each sample were sent to a commercial sequencing company (Inqaba Biotechnical Industries (Pty) Ltd, Pretoria, South Africa) for purification and sequencing in both directions. Quality of resultant sequences was assessed using Geneious Prime^®^ 2022.2.2 [32] (http://www.geneious.com) before consensus sequences were generated from both forward and reverse sequence reads for both fragments. A consensus sequence was then generated from both fragments. Sequences were identified using the Basic Local Alignment Search Tool (BLAST) (http://blast.ncbi.nlm.nih.gov/).

Phylogenetic analysis involved comparative sequences of species of *Dactylosoma*, *Karyolysus*, *Haemogregarina*, *Hemolivia* and *Hepatozoon* with *Adelina dimidiata*, *Adelina grylli* and *Klossia helcina* (GenBank: DQ096835, DQ096836, MT094865) as outgroup were downloaded from GenBank and aligned to the sequences generated within this study. Sequences were aligned using the MUSCLE alignment tool [33] implemented in Geneious Prime^®^ 2022.2.2. The alignment consisted of 60 sequences and was 1261 nt long. To infer phylogenetic relationships of the aligned dataset the Bayesian Inference (BI) method was used.

A model test was performed to determine the most suitable nucleotide substitution model using the Smart model selection software [34] (https://www.atgc-montpellier. fr/phyml-sms/). The best model identified was the General Time Reversible model with estimates of invariable sites and a discrete Gamma distribution (GTR + I + Γ). The BI analysis was implemented from within Geneious Prime® 2022.2.2 using MrBayes 3.2.2 [35]. The analysis was run twice over 10 million generations for the Markov Chain Monte Carlo (MCMC) algorithm, with a subsampling frequency of 200 generations and a ‘burn-in’ of 25%. Pairwise distances were generated by Mega-X 10.2.4 with a Kimura two-parameter substitution model with transitions and transversions included [36, 37], as in Zechmeisterová et al. [38].

## Results

**Fig. 1:**
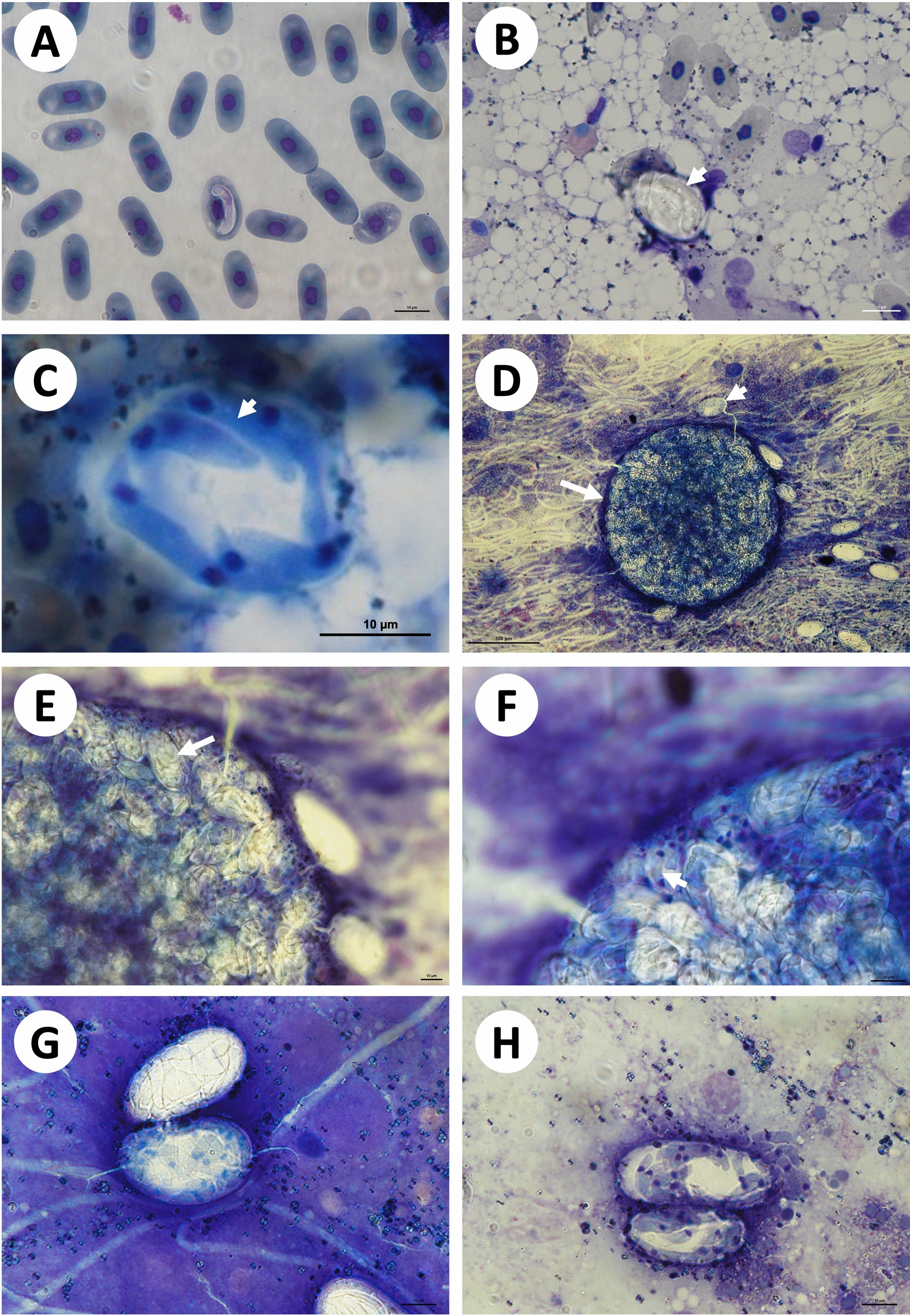
Peripheral blood, liver and tick stages. **A** Intraerythrocytic gamont in the peripheral blood of *Stigmocheylys pardalis* (3). **B**–**C** Meronts, with stain unabsorbed and absorbed, respectively, and **C** showing stained merozoites (arrowhead). **D**–**F** Oocyst in the haemocoel of *Amblyomma marmoreum*; **D** 200× magnification of mature sporulated oocyst containing numerous sporocysts (arrow), with surrounding free sporocysts (arrowhead), **E**–**F** 600× and 1000× magnification of oocyst respectively showing sporocyst (arrow), and **F** showing stained sporozoites within a sporocyst (arrowhead). **G**–**H** Free sporocysts in haemocoel, one unstained, others stained showing intrasporocystic sporozoites. Scale bar (**A**–**C**, **E**–**H**),10 µm, and (**D**), 100 µm

A total of 14 tortoises were collected, one *Kinixys natalensis* and one *Kinixys spekii*, seven *Kinixys zombensis* and five *Stigmochelys pardalis*. A total of 10 of the 14 (71%) tortoises were infested with ticks including larval, nymphal and adult stages. Larval and nymphal squashes yielded no identifiable haemogregarine stages and as such adult ticks formed the focus of this study. As species of *Kinixys* are often infected with both *H. fitzsimonsi* and *Hemolivia parvula*, three of the *S. pardalis* were selected for further analysis. Of these, one showed no evidence of haemogregarine peripheral blood stages [*S. pardalis* (1)], the second [*S. pardalis* (2)] showed an extremely low parasitaemia infection (0.1%) and the third [*S. pardalis* (3)] showed a relatively high parasitaemia (5%), of what was morphologically comparable with gamonts of *H. fitzsimonsi* (Fig. 1A). Within the third tortoise, the roadkill individual, merogonic stages were observed in the liver (Table 1). No observable stages were identified in the other organ smears.

**Table 1:** Summary of haemogregarine infection, tick species collected and screened for sporogonic stages, in and from three individual tortoises selected for analysis

*Stigmochelys pardalis* individual	Peripheral blood infection	Liver stages	*Amblyomma* species (*n*)	Oocyst	Sporocyst	Sporozoite
*S. pardalis* 1	−	−	*A. nuttalli* (1)	−	+	−
*S. pardalis* (2)	+	−	*A. herbraeum* (2)	−	+	+
*S. pardalis* (3)	+	+	*A. marmoreum* (1)	+	+	−

−, negative; +, positive; *n*, number

Three species of *Amblyomma* ticks (Acari; Ixodidae) were collected from the three *S. pardalis*; these were *Amblyomma herbraeum*, *Amblyomma marmoreum* and *Amblyomma nuttalli*. Impression slides from all three species showed sporogonic stages within the haemocoel. A single intact sporulated oocyst was observed in *A. marmoreum* (Table 1). No gametogenesis and subsequent fertilisation were observed in any of the ticks.

### Description of peripheral blood gamont and liver merogonic stages in *S. pardalis*

Gamonts were observed, measuring 16.2 ± 0.8 (15–17.3) × 2.7 ± 0.4 (1.8–3.2), with one broad pole, an opposite pole with a short, recurved tail, cytoplasm stained whitish-purple; nuclei 4.4 ± 0.8 (3–5.9) × 1.7 ± 0.3 (1.3–2.2) (*n* = 10) stained light purple, square or oval, were observed lying closer to the pole with the recurved tail (Fig. 1A).

Liver meronts were observed measuring 22.3 ± 2.1 (17.9–25.3) × 15.5 ± 1.1 (13.7–17.1) (*n* = 9) (Fig. 1B, C), often observed within a thick capsule inhibiting sufficient staining (Fig. 1B). Intrameront merozoites were vaguely visible as elongated structures when not stained. Stained meronts showed elongated merozoites and stained purple with a dark blue-purple staining nucleus (Fig. 1C). Attempts to count merozoites resulted in < 10 per meront (*n* = 9). No free merozoites were observed.

#### ***Remarks***

Gamont stages in the present study were morphologically comparable with those described by Dias [39] (15.84–18.81 × 3.63–5.61) and Cook et al. [3] (17–17.5 × 3.4–4.2). These were the only peripheral blood stages found in the present study. Meront stages were observed only in the liver, unlike that of other species of *Hepatozoon*, which may be observed also infecting various other internal organs such as the lungs [6, 13, 40, 41]. In the present study, the number of merozoites contained within meronts could not be conclusively determined, presumably due to the thick non-staining capsule. However, as numbers were low, this may suggest that they represent macromeronts [6]. Even so, the number of merozoites contained in macromeronts is highly variable among species of *Hepatozoon*. For instance, in species *Hepatozoon kisrae* Paperna, Kremer-Mecabell and Finkelman 2002 of the agamid *Laudakia stellio*, macromeronts divided into four to 16 macromerozoites [40], whilst in the species *Hepatozoon domerguei* Landau, Chabaud, Michel, and Brygoo 1970 of snakes, mature macromeronts could contain approximately 30 macromerozoites [6]. No cyst or cyst-like stages were observed in the present study, these stages often observed in the liver and lung tissue of snakes and lizards infected with *Hepatozoon*, such as in the case of *H. domerguei* [6].

### Description of sporogonic stages in unengorged *Amblyomma* spp. ticks

Mature, sporulated oocyst measured 126.1 × 120.7 (*n* = 1), spherical in shape, with a thin dark blue membrane, containing  > 100 oval sporocysts (Fig. 1D, arrow). Free in the haemocoel, surrounded by free sporocysts, some stained sporozoites were shown (Fig. 1D, arrowhead). Oocysts contained sporocysts visible under 60 × and 100 × magnification, and showed sporozoites, either unstained (Fig. 1E, arrow) or stained with light blue cytoplasm and visible nuclei stained dark blue (Fig. 1F, arrowhead), respectively.

**Fig. 2:**
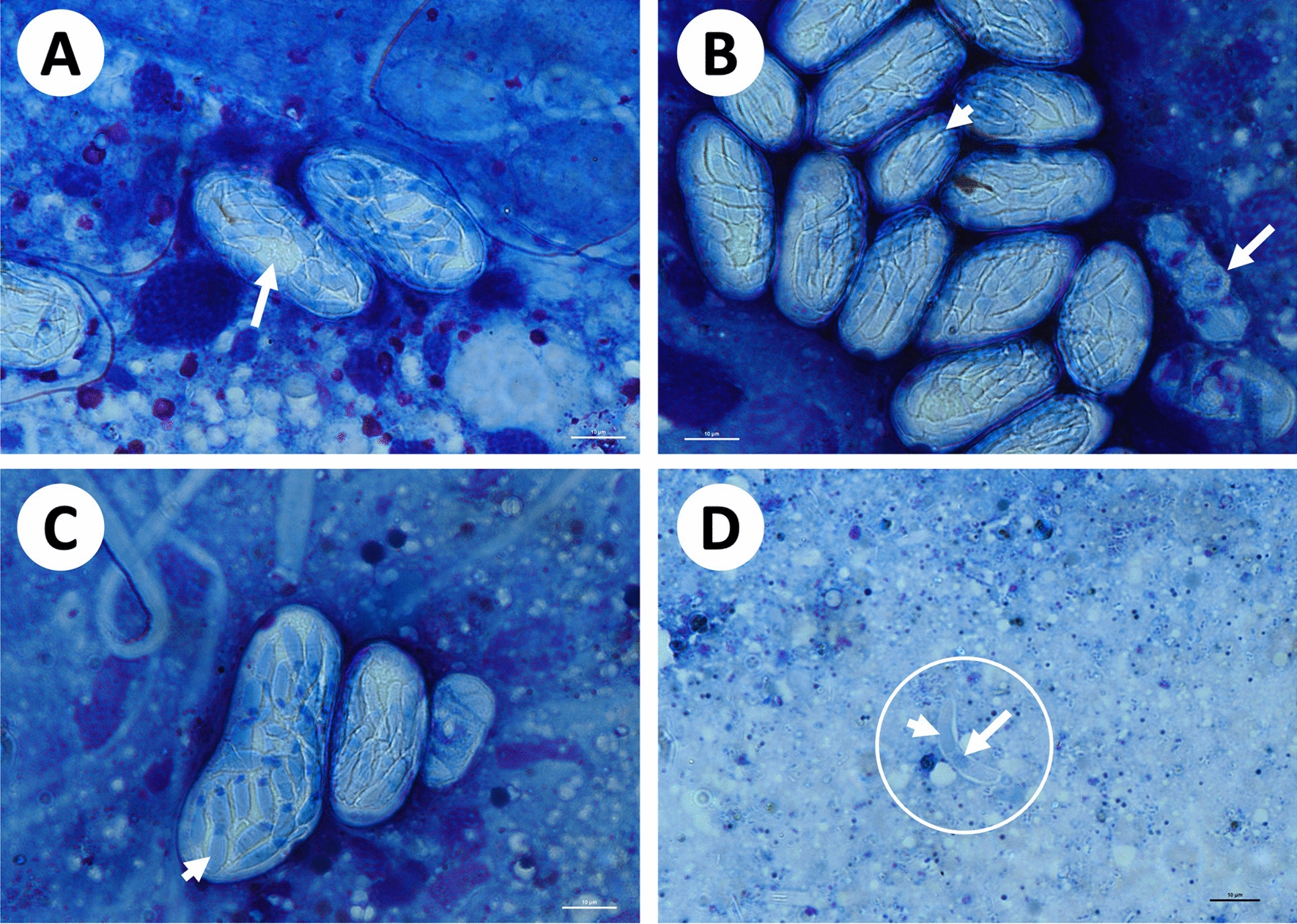
Sporogonic stages in the haemocoel of *Amblyomma nuttalli* and *Amblyomma herbraeum* collected from *Stigmochelys pardalis* (1) and (2), respectively. **A** Sporocyts in *A. nuttalli*, one showing developed sporozoites, the other showing a central residual body (arrow). **B**–**D** Sporocysts and sporozoite in *A. herbraem*, **B** showing larger and smaller more ovoid sporocyst (arrowhead), and either sporocysts prior to division or in the process of degrading (arrow), and **C** showing a mature sporocyst with developed sporozoites (arrowhead), a developing/maturing and undeveloped sporocyst. **D** Sporozoite (circled), surrounded by a clear membrane, showing a nucleus (arrow) and potential crystalline body (arrowhead). Scale bar, 10 µm

Sporocysts measured 38.2 ± 5.6 (29.2–49.5) × 19.6 ± 2.7 (14–24.7) in *A. marmoreum* (*n* = 15) and contained ~18 sporozoites (*n* = 7) (Fig. 1G–H), either unstained (Fig. 1G) or stained (Fig. 1G–H) and the latter sporozoites had blue-staining cytoplasms and blue to dark blue staining nuclei and some sporozoites were seen exiting the sporocyst (Fig. 1H). There was variability in size of sporocysts, with one larger than the other (Fig. 1H). Sporocysts measuring 37.2 ± 3.0 (29.9–43.4) × 17.8 ± 1.9 (14.9–21.9) in *A. nuttalli* (*n* = 26) contained ~ 20 sporozoites (*n* = 4) (Fig. 2A). A maturing sporocyst showed basal mass or residual body at the centre (Fig. 2A, arrow). Sporocysts measuring 33.8 ± 3.3 (29–40.1) × 17.5 ± 2.5 (13.7–20.9) in *A. herbraeum* (*n* = 20) contained~ 21 sporozoites (*n* = 3) (Fig. 2B, C), showing smaller, more spherical than narrowly to broadly oval sporocysts (Fig. 2B, arrowheads) and potentially degraded sporocysts or those prior to division (Fig. 2B, arrow); in some cases mature sporocysts with well-differentiated sporozoites (Fig. 2C, arrowhead) were much larger than a maturing sporocyst and a potentially undeveloped sporocyst to the right. All sporocysts with a thin, often white or blue-stained capsule.

Sporozoites measured 10.2 ± 1.0 (8.7–11.8) × 1.7 ± 0.3 (1.3–2.1) (*n* = 12), with the nucleus measuring 1.4 ± 0.3 (0.8–1.7) × 1.0 ± 0.2 (0.7–1.4) (*n* = 10), in *A. herbraeum* (Fig. 2D), and were elongated and slender, slightly curved with blue-staining cytoplasm and darker blue-staining central nucleus that was rounded in shape (Fig. 2D, arrow). A denser elongated oval structure on one side of the nucleus, potentially representing a crystalline body, was observed (Fig. 2D, arrowhead).

#### ***Remarks***

In the present study, sporogonic stages were observed free within the tick haemocoel, typical of species of *Hepatozoon* and unlike that of *Hemolivia*, which is found within the intestinal cells [1, 6]. The single mature sporulated oocyst, containing numerous sporocysts was spherical in shape, similar to that found with oocysts of the tick hosted *Hepatozoon tuatarae* (Laird 1950) of tuatara. Compared with *H. tuatarae* and another tick-hosted species of *Hepatozoon*, *H. kisrae*, the oocyst of *H. fitzsimonsi* was half the size 126.1 × 120.7 as compared with 236 × 228 and 200–230 × 230, respectively [6, 40, 42]. Difference in size was also noted in sporocyst stages, those in the present study being longer (mean 33–38) than those of *H. tuatarae* (22.4), but containing similar numbers of sporozoites, namely the present study being 18–21 and those of *H. tuatarae* being 18. In comparison with sporocysts of *H. kisrae* (34 × 24–61 × 23), the present study’s sporocysts were on average smaller (mean 33–38 × 18–20) and different in shape, namely ovoid compared with the ellipsoidal sporocysts of *H. kisrae* [6], with fewer sporozoites (*H. kisrae* 16–35). Sporozoite dimensions were not provided for *H. tuatarae* as none were found free of the sporocysts [42], nor for *H. kisrae* [6, 40]. Interestingly, sporocyst stages in this study were longer and much wider than those described by Cook et al. [3], measuring 33–38 × 18–20 as compared with 26–30 × 9–13, respectively. Sporozoite numbers contained in sporocysts, however, were similar, namely 18–21 in this study as compared with 16–18 in Cook et al. [3]. Sporozoites in both studies were similar in size, measuring 10.2 × 1.7 in the present study as compared with 13.6 × 1.9 in Cook et al. [3]. In both studies, sporozoites were slightly curved or banana shaped as was seen with *H. tuatarae*. In Cook et al. [3], sporozoites appeared to have two purple stained structures on either side of the nucleus, suggested at the time to be crystalline bodies. Only one of these structures were observed in the sporozoites in this study.

### Phylogenetic analysis of blood and tick stages

A 1733 nt and 1688 nt sequence was achieved for the *Hepatozoon* infecting *S. pardalis* (2) and *S. pardalis* (3) (GenBank accession numbers: PP718267, PP718268 respectively). A 626 nt sequence was isolated from *A. nuttalli* collected from *S. pardalis* (1), along with a 620 and 626 nt sequences for *A. herbraeum* collected from *S. pardalis* (2) (GenBank accession numbers: PP718266, PP718263, PP718264 respectively). From *A. marmoreum*, collected from *S. pardalis* (3), a 994 nt sequence was isolated (GenBank accession number: PP718265). Between all sequences isolated from these ticks and the blood of tortoises, genetic divergences were 0%, strongly suggesting that the blood stages and those in the ticks represent the same haemogregarine species.

**Fig. 3:**
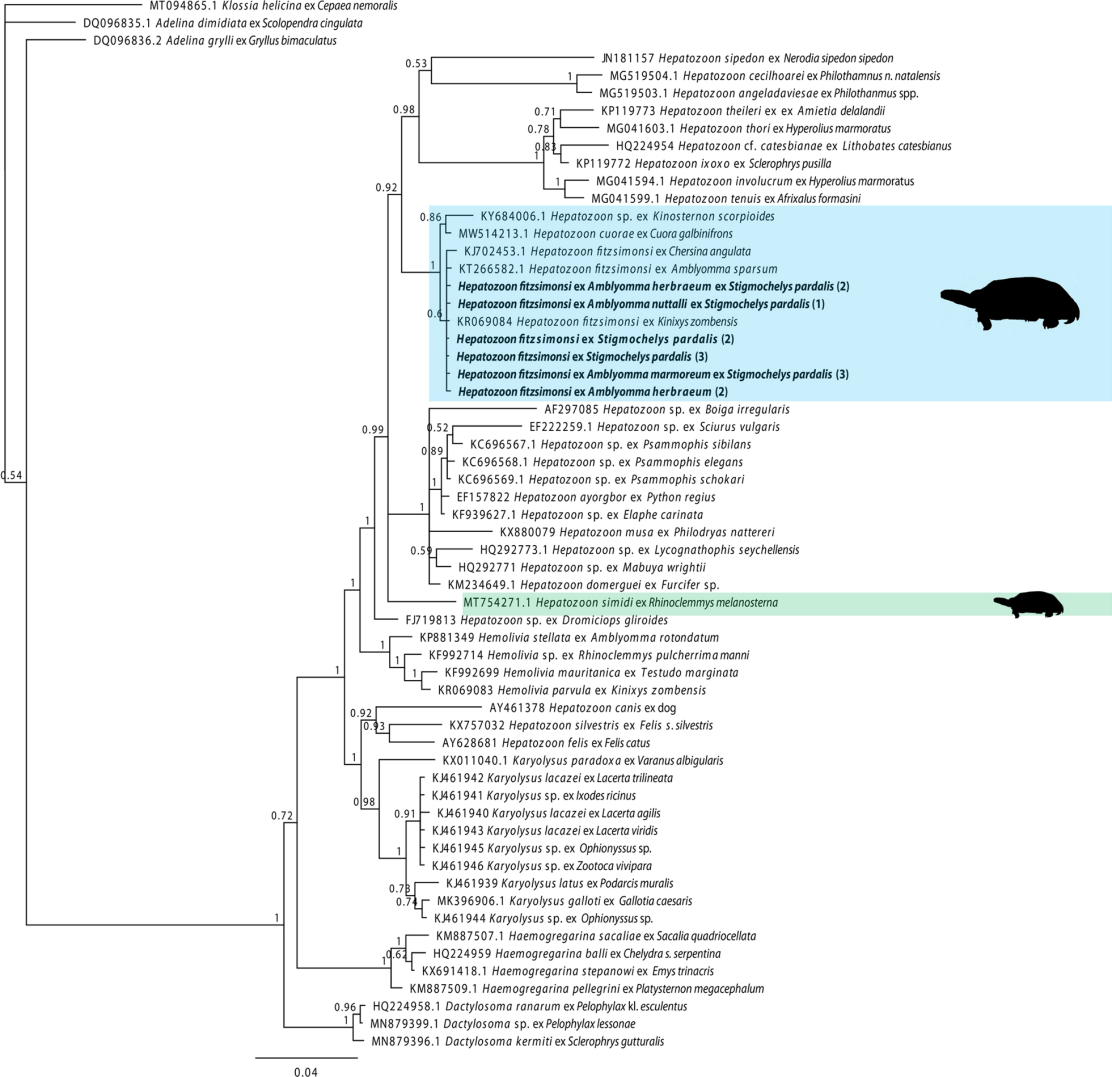
Phylogenetic tree of haemogregarine partial 18S rDNA sequences (60 sequences and 1261 nt) based on Bayesian inference (BI) analysis. Nodal supports shown as Bayesian posterior probabilities. Blue clade showing species of *Hepatozoon* of chelonians, including *Hepatozoon fitzsimonsi* from this (bolded) and previous studies. Green sequence is that of *Hepatozoon simidi* isolated from a terrapin species from Brazil. It is the only *Hepatozoon* not included in the main chelonian *Hepatozoon* clade. Outgroup comprised *Adelina dimidiata*, *Adelina grylli* and *Klossia helcina* (GenBank: DQ096835, DQ096836, MT094865). The scale bar represents 0.04 nucleotide substitutions per site

Comparisons of those isolates from the current blood stage material [*S. pardalis* (2) and (3)] with previous molecular descriptions of *H. fitzsimonsi* (KJ702453, KR069084), showed a genetic divergence of 0–0.2% (Table 2). This was the same for the isolates sequenced from the ticks in the current study; the blood and tick isolates clustered in a monophyletic clade consisting of the known *H. fitzsimonsi* (Fig. 3). Genetic divergence was 0% for that collected from both tortoise blood and ticks with *A. sparsum* collected off tortoises in Kenya, clustering within the same *H. fitzsimonsi* clade. *Hepatozoon cuorae* (Chai and Chen, 1990) [38, 43] showed the closest relationship with the isolates from this study, with a divergence of 0.2–0.6% (Table 2). The *Hepatozoon* sp. sequence from *Kinosternon scorpioides* showed variable divergence (0.2–1.1%) between sequences of this study and known *H. fitzsimonsi* (Table 2), which was dependent on length of sequence and coverage. Both *H. cuorae* and the latter *Hepatozoon* sp. clustered as sister taxa to the *H. fitzsimonsi* clade. Divergence between *Hepatozoon simidi* Gutiérrez-Liberato, Lotta-Arévalo, Rodríguez-Almonacid, Vargas-Ramírez, and Matta 2021 [44] and the current material was 3%, clustering separately and basal to a clade containing *Hepatozoon* of squamata (Fig. 3).

**Table 2:** Genetic distances between species of *Hepatozoon* infecting chelonians with species of *Hemolivia* as reference for genetic distance between genera. Sequences 9–14 are those of the current study

		1	2	3	4	5	6	7	8	9	10	11	12	13	14
1	KF992699 *Hemolivia mauritanica* ex *Testudo marginata*														
2	KR069083 *Hemolivia parvula* ex *Kinixys zombensis*	0.006													
3	MT754271 *Hepatozoon simidi* ex *Rhinoclemmys melanosterna*	0.035	0.035												
4	KY684006 *Hepatozoon* sp. ex *Kinosternon scorpioides*	0.048	0.021	0.016											
5	KJ702453 *Hepatozoon fitzsimonsi* ex *Chersina angulata*	0.035	0.036	0.023	0.003										
6	MW514213 *Hepatozoon cuorae* ex *Cuora galbinitrons*	0.031	0.020	0.028	0.009	0.004									
7	KT266582 *Hepatozoon fitzsimonsi* ex *Amblyomma sparsum*	0.033	0.031	0.045	0.007	0.003	0.002								
8	KR069084 *Hepatozoon fitzsimonsi* ex *Kinixys zombensis*	0.024	0.021	0.030	0.009	0.002	0.002	0.000							
9	*Hepatozoon fitzsimonsi* ex *Stigmochelys pardalis* (2)	0.029	0.020	0.030	0.011	0.002	0.006	0.000	0.000						
10	*Hepatozoon fitzsimonsi* ex *Amblyomma herbraeum* ex *Stigmochelys pardalis* (2)	0.028	0.028	0.030	0.002	0.002	0.002	0.000	0.000	0.000					
11	*Hepatozoon fitzsimonsi* ex *Amblyomma nuttalli* ex *Stigmochelys pardalis* (1)	0.028	0.028	0.030	0.002	0.002	0.002	0.000	0.000	0.000	0.000				
12	*Hepatozoon fitzsimonsi* ex *Stigmochelys pardalis* (3)	0.029	0.020	0.030	0.011	0.002	0.005	0.000	0.000	0.000	0.000	0.000			
13	*Hepatozoon fitzsimonsi* ex *Amblyomma herbraeum* (2)	0.028	0.028	0.030	0.002	0.002	0.002	0.000	0.000	0.000	0.000	0.000	0.000		
14	*Hepatozoon fitzsimonsi* ex *Amblyomma marmoreum* ex *Stigmochelys pardalis* (3)	0.025	0.022	0.030	0.009	0.002	0.002	0.000	0.000	0.000	0.000	0.000	0.000	0.000

#### ***Remarks***

As the isolates of the present study cluster primarily with known *H. fitzsimonsi* and within a larger clade containing *Hepatozoon* of other chelonians, those isolates from unengorged ticks containing sporogonic stages are considered representative of *H. fitzsimonsi*. Where a divergence of 0.2% was noted between sequences of *H. fitzsimonsi*, this would be because of comparisons between shorter and longer sequences. Sequences from ticks were shorter than desired as the primer set HepF300 and HepR900 was used instead of the primer sets and protocol used for the tortoise blood (which produced longer sequences). However, this primer set does amplify a fragment of a highly variable region in the 18SrRNA gene and is therefore adequate for phylogenetic analysis [38]. Alignment of the present study’s sequences and those of known *H. fitzsimonsi* were identical but for one base pair in one sequence, when comparing this region, confirming the haemogregarine within the ticks to be *H. fitzsimonsi*. The low support for this clade is therefore presumed to result from low evolutionary signal or variation among the sequences. Comparisons of *H. fitzsimonsi* from tortoise blood to *H. cuorae* showed a divergence of 0.5–0.6% for comparatively long sequences (*H. cuorae*: 1715 nt; *H. fitzsimonsi* tortoise 2 and 3: 1733 nt and 1688 nt, respectively), confirming that they are separate species whilst taking into consideration the conservative nature of the 18S rRNA gene. Regarding the placement of *H. simidi* with *Hepatozoon* spp. of squamata, this too was observed by Zechmeisterová et al. [38], where this species was observed in these authors’ phylogenetic analysis with a *Hepatoon* sp. from a snake.

## Discussion

*Hepatozoon fitzsimonsi* was the first chelonian haemogregarine to be reassigned to the genus *Hepatozoon* on the basis of morphological and molecular findings [3]. In the years that followed, two other species of *Hepatozoon* were described and reassigned from chelonians, *Hepatozoon simidi* and *Hepatozoon cuorae*, respectively [38, 44]. Further reports for *Hepatozoon* in chelonians include an unpublished speculative sequence of *H. fitzsimonsi* from an unnamed *Kinixys* sp. from Nigeria, a sequence of *H. fitzsimonsi* from the tick *Amblyomma sparsum* from tortoises from Kenya [11, 16, 38], and the *Hepatozoon* sp. reported from *Gopherus polyphemus* [45] and *Sternotherus odoratus* [46] in the USA. As mentioned by Zechmeisterová et al. [38], two sequences, named as *Hepatozoon* on GenBank, from *Mauremys leprosa* by Marzal et al. [47], are in fact representatives of *Haemogregarina*.

For the three morphologically described and molecularly supported species of chelonian *Hepatozoon* above, the only species for which a potential definitive host has been provided is *H. fitzsimonsi* [3, 38, 44]. The present study found sporogonic stages in three tick species of the genus *Amblyomma*, which were morphologically comparable with those found in ticks by Cook et al. [3], linking these molecularly to *H. fitzsimonsi*. Sporocyst stages in the present study were on average longer and much wider than those described in Cook et al. [3]; this was particularly evident when observing the range in length and width of sporocysts. Simultaneously, 61 sporocysts were measured in the present study as compared with the 10 in Cook et al. [3]. Furthermore, sporocysts were observed in the present study in what appeared to be various stages of development: some were mature and contained developed sporozoites, others had a residual body still present, and others were smaller and more spherical. At times sporocysts were seen which appeared to have no sporozoite development within and, equally, sporozoites were seen that appeared to be degrading or observed just prior to division, the former potentially representing an older phase of infection. The variability in size and shape of sporocysts, besides being a potential result of development phase, appears to be typical in *Hepatozoon* between species [13] and is also evident within species – *Hepatozoon rarefaciens* (Sambon and Seligmann, 1907) [48] (21 × 21–59 × 59), *Hepatozoon fasciatae* Telford, Wozniak and Butler 2001 [49] (15–45 × 14–30), *Hepatozoon seminatrici* Telford, Wozniak and Butler 2001 [49] (30–56 × 23–34), *Hepatozoon sistruri* Telford, Butler and Telford 2002 [50] (25–50 × 20–50), and *Hepatozoon ayorgbor* Sloboda, Kamler, Bulantová, Votýtpka and Modrý 2007 [41] (20–53 × 18–42) [6]. Two purple-stained structures were seen on either side of the nucleus of sporozoites in the material of Cook et al. [3], which were not seen in the present study’s sporozoites; however, this may be as a result of staining bias. Regardless, as sequencing of tick stages in Cook et al. [3] was unsuccessful and thus it cannot be ascertained that the infection seen in this previous study was representative of *H. fitzsimonsi*.

Only a single intact oocyst was observed in the material of the present study, regardless of the care taken to be as gentle as possible when preparing the impression slides. This, and the thin oocyst wall, suggests that these stages rupture readily, as in *Hepatozoon clamatae* Stebbins [51] of frogs. Similarly, as with sporocysts, oocysts too show a high variability in dimensions between species [13]; however, as only one oocyst was found, it cannot be determined if this may also be at an intra-species level.

Meronts observed in the liver of the one tortoise were tentatively identified as macromeronts. No larger meronts containing higher numbers of merozoites, which may have represented micromeronts, were observed in the current material. Macromeronts are considered the first stage of merogonic development in species of *Hepatozoon*, which eventually give rise to micromeronts, the micromerozoites of which will in time enter erythrocytes to form gamonts [13]. As only mature gamonts were observed in the peripheral blood of the tortoise, with no younger stages such as trophozoites, along with the lack of probable micromeronts in the liver, it may be suggested that these indicate two separate infections. Both macro- and micromeronts have not been observed for any species of *Hemolivia* so far [52], including *Hemolivia mauritanica*, a species infecting terrestrial tortoises of the western palearctic realm [52, 53]. *Hemolivia parvula* (Dias, 1953) has been found to infect terrestrial tortoises of eastern southern Africa. However, naturally it appears to infect only species of *Kinixys*, with only a single finding in a captive *S. pardalis* [3, 15, 54]. Regardless, as the risk of co-infection, particularly in chelonian hosts, is high, as is evident in the study by Zechmeisterová et al. [38], care needed to be taken that stages seen in both tortoise and tick hosts were not that of *Hm. parvula*. As such, during the analyses of the raw sequence data, chromatograms were checked for evidence of double peaks, which would be suggestive of co-infections. As no double peaks were observed and the quality of sequences was high, it supports that the merogonic stage seen in the liver of the present material is most likely that of *H. fitzsimonsi* and not *Hm. parvula*. Compared with other species of *Hepatozoon* and species of *Hemolivia*, in which cystic stages are often present [6, 13, 52], no cystic stages were observed in the current study. It has been suggested that these stages aid in the persistence of infection [6, 52] and may play a role in transmission of infection from one vertebrate to another, particularly in three host life cycles [6]. However, these scenarios require further investigation at a more individual level and cannot be considered a general rule.

Karadjian et al. [12] suggested that *H. fitzsimonsi* be reassigned as a species of the then newly erected genus *Bartazoon*, which is transmitted by biting insects. Even though infection of more than one group of arthropod hosts cannot be ruled out at this stage, particularly given the findings on *Hepatozoon fusifex* Ball et al. [55], which showed infection in its natural tick host along with experimentally infected mosquitoes of widely different origin [6], it appears rare. For instance, in the dissection of 100 ticks feeding on *H. ayorgbor*-infected snakes, only the mosquitoes were found to contain sporogonic stages [6, 41]. Equally, attempts by Morsy et al. [56] to detect sporogony of the snake haemogregarine species *Hepatozoon seurati* (Laveran and Pettit 1911) in mites, ticks, sand flies and *Aedes aegypti* fed on infected snakes, were unsuccessful with stages found only in epidemic *Culex pipiens molestus* [6, 56]. This was equally true for *Hepatozoon tupinambis* (Laveran and Salimbeni 1909), where attempts to infect leeches and triatomid bugs on infected teiid lizards was unsuccessful [6]. Given that sporogonic stages, with the final infective developmental stage, the sporozoite, has been identified in unengorged *Amblyomma* ticks and linked molecularly to *H. fitzsimonsi* in this study, it is assumed that these or at least three species of this genera can act as definitive hosts and vectors for *H. fitzsimonsi.* However, the transmission capability of these ticks will need further investigation, as well as to how tortoises become infected. Tortoises do display aggressive behaviour during courtship or when competing for a mate, often biting the other tortoise. This can be observed in Desert tortoises in the USA, as well as in tortoise species in South Africa [57], Cook, personal observation]. It may be in such cases where ingestion of infected ticks occurs. If species of *Amblyomma* are vectors for *H. fitzsimonsi*, which these findings highly suggest that they are, *H. fitzsimonsi* does not fit within the taxonomic description of *Bartazoon* (transmitted only by biting insects), making the genus paraphyletic.

## Conclusions

As mentioned previously, *H. fitzsimonsi* was the first haemogregarine of chelonians to be reassigned from *Haemogregarina* to *Hepatozoon* on the basis of on morphological and molecular findings. This was particularly significant as it broadened the range of potential vectors for this species and was therefore no longer limited to leeches. Even though Cook et al. [3] did find sporogonic stages in ticks associated with tortoises infected with *H. fitzsimonsi*, these parasites could not be confirmed as definitive hosts. In the present study sporogonic stages in three species of *Amblyomma* ticks were molecularly confirmed to be that of *H. fitzsimonsi*, substantiating that these parasites do act as definitive hosts and strongly suggesting that they are a vector. Even though further research into the transmission of *H. fitzsimonsi* from ticks to tortoises may be required, this would be the second haemogregarine of tortoises for which ticks are the vector, with *Hemolivia mauritanica* being the first. Importantly, this is the first species of *Hepatozoon* infecting chelonians for which ticks have been identified as a definitive host. This will hopefully encourage further research, identifying definitive hosts and vectors for other species of chelonian *Hepatozoon*, a research area that remains largely neglected at present.

## Data Availability

Data, including sequences generated, that support the findings of this study, are available in the manuscript and on the GenBank database under accession nos. PP718263–PP718268.
